# NRF2 Enables EGFR Signaling in Melanoma Cells

**DOI:** 10.3390/ijms22083803

**Published:** 2021-04-07

**Authors:** Julia Katharina Charlotte Kreß, Christina Jessen, André Marquardt, Anita Hufnagel, Svenja Meierjohann

**Affiliations:** 1 Institute of Pathology, University of Würzburg, 97080 Würzburg, Germany; julia.kress@uni-wuerzburg.de (J.K.); christina.jessen@biozentrum.uni-wuerzburg.de (C.J.); andre.marquardt@uni-wuerzburg.de (A.M.); anita.hufnagel@uni-wuerzburg.de (A.H.); 2 Comprehensive Cancer Center Mainfranken, University of Würzburg, 97080 Würzburg, Germany

**Keywords:** EGFR, NRF2, NFE2L2, KEAP1, MITF-low, TGF-alpha, EGF, NSCLC, HNSC

## Abstract

Receptor tyrosine kinases (RTK) are rarely mutated in cutaneous melanoma, but the expression and activation of several RTK family members are associated with a proinvasive phenotype and therapy resistance. Epidermal growth factor receptor (EGFR) is a member of the RTK family and is only expressed in a subgroup of melanomas with poor prognosis. The insight into regulators of EGFR expression and activation is important for the understanding of the development of this malignant melanoma phenotype. Here, we describe that the transcription factor NRF2, the master regulator of the oxidative and electrophilic stress response, mediates the expression and activation of EGFR in melanoma by elevating the levels of EGFR as well as its ligands EGF and TGFα. ChIP sequencing data show that NRF2 directly binds to the promoter of EGF, which contains a canonical antioxidant response element. Accordingly, EGF is induced by oxidative stress and is also increased in lung adenocarcinoma and head and neck carcinoma with mutationally activated NRF2. In contrast, regulation of *EGFR* and *TGFA* occurs by an indirect mechanism, which is enabled by the ability of NRF2 to block the activity of the melanocytic lineage factor MITF in melanoma. MITF effectively suppresses *EGFR* and *TGFA* expression and therefore serves as link between NRF2 and EGFR. As EGFR was previously described to stimulate NRF2 activity, the mutual activation of NRF2 and EGFR pathways was investigated. The presence of NRF2 was necessary for full EGFR pathway activation, as NRF2-knockout cells showed reduced AKT activation in response to EGF stimulation compared to controls. Conversely, EGF led to the nuclear localization and activation of NRF2, thereby demonstrating that NRF2 and EGFR are connected in a positive feedback loop in melanoma. In summary, our data show that the EGFR-positive melanoma phenotype is strongly supported by NRF2, thus revealing a novel maintenance mechanism for this clinically challenging melanoma subpopulation.

## 1. Introduction

Cutaneous melanomas are mainly driven by oncogenic mutations in BRAF, NRAS, and NF1, which efficiently activate the MEK/ERK1/2 pathway [1,2]. As an overactivation of the pathway is deleterious for cancer cells, mutations in these oncogenes usually exclude each other and only occur under therapy stress, e.g., when the pathway is blocked by BRAF and/or MEK inhibitors [1,2,3,4,5,6,7,8,9]. Furthermore, the activity of receptor tyrosine kinases including EGFR, another efficient MEK/ERK1/2 activator, is suppressed in melanoma under normal circumstances. This is in part attributed to the ERK1/2-driven expression of the SPRED and SPRY feedback inhibitors, which block EGFR/RAS signaling [10,11]. EGFR expression is therefore only detectable in a subset of melanoma cells [12]. However, if expressed, EGFR correlates with melanoma invasiveness and pro-metastatic features [13,14]. Several melanoma models support the pro-metastatic role of EGFR, as it enhances cellular migration as well as angiogenesis [15,16,17]. Like the proinvasive receptor tyrosine kinase AXL, EGFR is expressed in dedifferentiated melanoma cells in a negative correlation with the melanocytic lineage factor MITF [12]. EGFR was furthermore reported to mediate resistance to BRAF inhibitors in melanoma [18,19], and the high EGFR expression in colorectal cancer cells is considered to be the reason for the lack of BRAF inhibitor responsiveness of BRAF^V600E^ mutant colorectal cancer [20,21,22].

In melanoma, elevated EGFR activity is mostly caused by transcriptional regulation, but not by the appearance of oncogenic EGFR mutations. The understanding of the conditions enabling the generation of this EGFR^high^ melanoma subpopulation therefore has a high biological relevance for this tumor entity.

In a study addressing the function of the transcription factor Nuclear Factor Erythroid 2 Like 2 (NFE2L2 or more common NRF2) in melanoma, we observed that *EGFR* is strongly reduced after NRF2 knockdown [23]. NRF2 is a short-lived protein, which is kept in check by its cytosolic interaction partner Kelch-like ECH-associated protein 1 (KEAP1) in absence of oxidative stress. KEAP1 mediates the interaction of NRF2 with the cullin 3 (CUL3)/RBX ubiquitin ligase complex and thereby facilitates its degradation [24,25]. Under certain stress conditions, including oxidative stress or autophagy deregulation, the interaction between KEAP1 and NRF2 is weakened, and NRF2 can accumulate in the nucleus and induce genes involved in antioxidant stress defense and metabolism [26,27,28]. Stress adaptation by NRF2 enables cancer cells to cope with challenging conditions in the tumor microenvironment, such as limited nutrient supply or oxidative stress from tumor-infiltrating immune cells, and is therefore responsible for resilience and therapy resistance in several cancer entities [29].

Here, we describe that NRF2 is also involved in maintaining the EGFR-expressing subgroup of melanoma cells. By directly inducing *EGF* via an ARE promoter element and derepressing *EGFR* and *TGFA*, NRF2 enables EGFR activation as well as its responsiveness to EGFR ligands. Reversely, EGFR also activates NRF2, resulting in a feed-forward loop with joint activation of EGFR and NRF2 pathways.

## 2. Results

### 2.1. Regulation of EGFR and EGFR Ligands by NRF2

**Figure 1:**
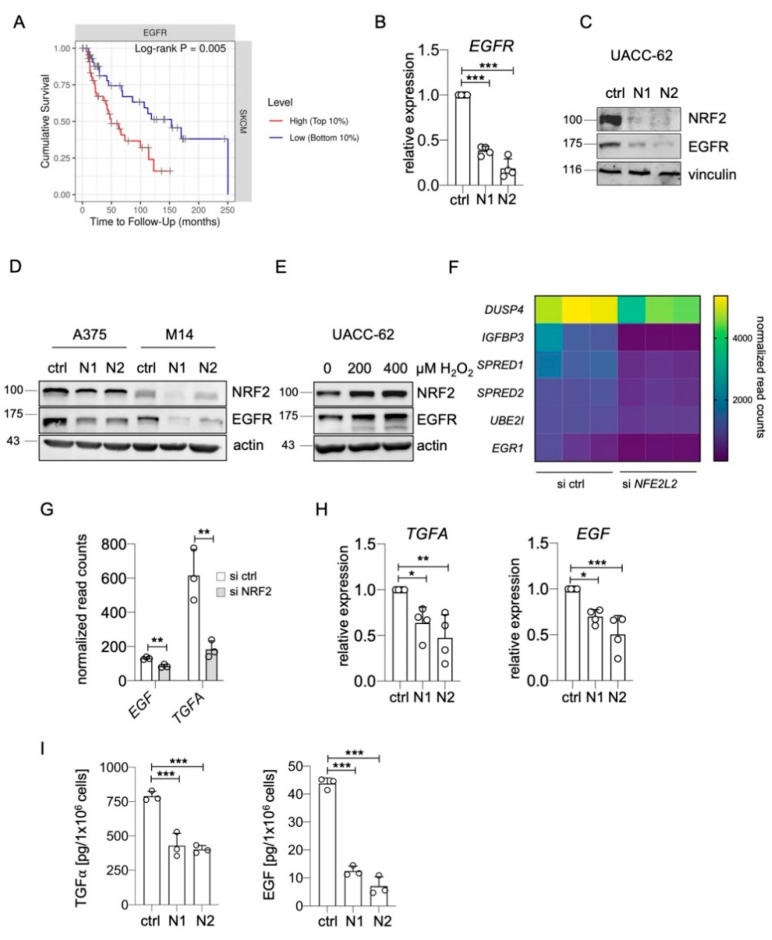
**Regulation of EGFR and EGFR ligands by NRF2.** (**A**) Kaplan–Meier plot of cumulative survival in SKCM melanomas, split into two groups with the 10% highest and the 10% lowest *EGFR* expression (https://cistrome.shinyapps.io/timer/ (accessed on 22 March 2021)). (**B**) Real-time PCR of *EGFR* gene expression after siRNA-dependent knockdown of *NFE2L2* in UACC-62 cells (3 d). Two independent siRNAs against *NFE2L2* are termed “N1” and “N2”. One-way ANOVA with Dunnett’s multiple comparisons test was carried out to calculate significant differences. Error bars represent SD. (**C**) Immunoblot showing NRF2 and EGFR expression after siRNA-dependent knockdown of *NFE2L2* in UACC-62 cells (3 d). Vinculin served as loading control. (**D**) Immunoblot showing NRF2 and EGFR expression after siRNA-dependent knockdown of *NFE2L2* in A375 and M14 cells. Actin served as loading control. (**E**) Immunoblot of NRF2 and EGFR expression in response to H_2_O_2_ treatment (200 or 400 µM, 5 h). Actin served as loading control. (**F**) Differential expression of EGFR pathway target genes in UACC-62 melanomas cells treated with control or *NFE2L2*-specific siRNA. Data are derived from previously published transcriptome data [23] (Bioproject accession number PRJNA601317), and normalized read counts were analyzed in GraphPadPrism (padj < 0.05). (**G**) *EGF* and *TGFA* expression, derived from the same dataset as subfigure (F). Two-tailed Student’s *t*-test was carried out to calculate significant differences. (**H**) Real-time PCR of *EGF* and *TGFA* gene expression after siRNA-dependent knockdown of *NFE2L2* in UACC-62 cells (3 d). One-way ANOVA with Dunnett’s multiple comparisons test was carried out to calculate significant differences. (**I**) TGFα and EGF ELISA done with UACC-62 cells after siRNA-dependent knockdown of *NFE2L2* and compared to control siRNA-treated cells. One-way ANOVA with Dunnett’s multiple comparisons test was carried out to calculate significant differences. (* *p* < 0.05, ** *p* < 0.01, *** *p* < 0.001).

A fine balance of cellular pro- and antioxidants is required to enable proliferation and survival, particularly in tumors such as melanoma, which encounter numerous sources of reactive oxygen species (ROS) [30,31,32]. The ROS-inducible transcription factor NRF2 is a main determinant of this balance, as it induces the transcription of genes involved in glutathione and thioredoxin synthesis and regeneration [29]. In a recent RNA sequencing experiment of UACC-62 melanoma cells treated with control or *NFE2L2*-specific siRNA, we observed that *EGFR* belongs to the top 10 genes with the strongest suppression under conditions of NRF2 depletion [23]. As high *EGFR* expression goes along with a poor prognosis in skin cutaneous melanomas (Figure 1A), we further analyzed the effect of NRF2 on EGFR regulation. Using two independent siRNAs against *NFE2L2*, we detected a strong reduction in EGFR RNA and protein expression in NRF2-depleted UACC-62 cells (Figure 1B,C). The positive effect of NRF2 on EGFR levels was confirmed in two independent EGFR-positive melanoma cell lines, where the extent of EGFR reduction notably correlated with the efficiency of the *NFE2L2* knockdown (Figure 1D). Furthermore, when NRF2 levels were elevated by H_2_O_2_ treatment, this resulted in higher EGFR levels (Figure 1E).

Next, we went back to the RNA sequencing data to test if the expression of target genes of the EGFR signaling pathway [33] was also affected by the *NFE2L2* knockdown. A reduction of several EGFR downstream genes was indeed detected (Figure 1F), indicating that NRF2 might also increase the activity of the EGFR pathway.

As mutant NRF2 was previously described to enhance the expression of EGFR ligands *Tgfa*, *Areg*, and *Egf* in a mouse model for hepatomegaly [34], we considered the possibility that endogenous wild type NRF2 might have a similar effect in human melanoma. Among the seven EGFR ligands [35], *EGF* and *TGFA* were expressed in UACC-62 cells and were downregulated after *NFE2L2* knockdown (Figure 1G). This regulation was confirmed by real-time PCR (Figure 1H). To test the functional impact of these observations, TGFα and EGF ELISA assays were conducted, using the supernatant of UACC-62 cells. Both EGFR ligands were secreted from UACC-62 cells, and this secretion was strongly reduced when NRF2 was knocked down by siRNA (Figure 1I).

**Figure 2:**
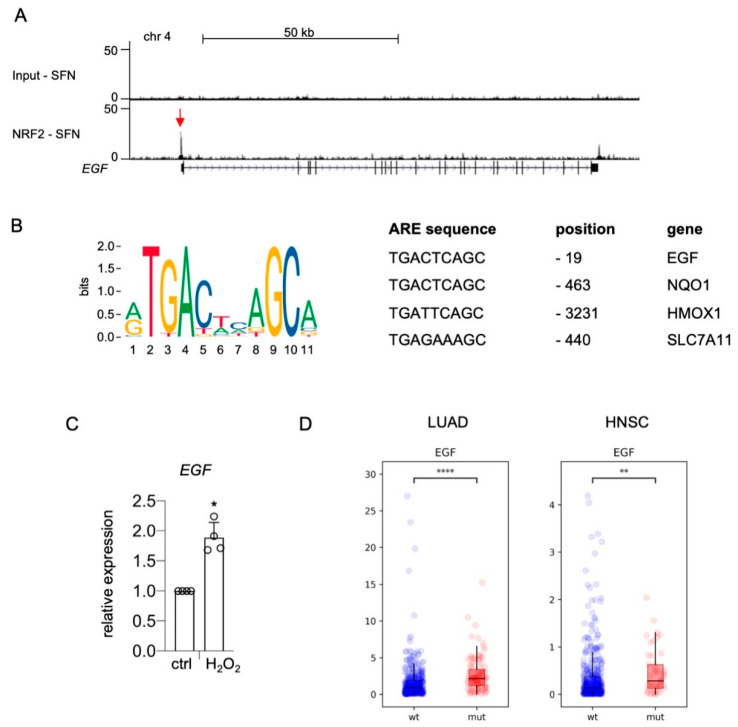
**Direct binding of NRF2 to the EGF promoter.** (**A**) Genome browser tracks of the *EGF* promotor region bound by NRF2, evaluated by ChIP-Seq analysis [23]. NRF2 was stabilized by treatment with sulforaphane (7.5 µM SFN, 24 h). (**B**) Left: Consensus NRF2 binding ARE sequence, according to the matrix profile from JASPAR 2020 (http://jaspar.genereg.net (accessed on 22 March 2021)) [38]. Right: Comparison of ARE sequences in the promoter region of the indicated human genes. Information about the *EGF* promoter is from the present study, while *NQO1*, *HMOX1*, and *SLC7A11* information is from [39]. (**C**) Real-time PCR of *EGF* expression in response to H_2_O_2_ (400 µM, 5 h). Two-tailed Student’s *t*-test with Mann–Whitney test was carried out to calculate significant differences (* *p* < 0.05). (**D**) RNA expression of *EGF* in TCGA lung adenocarcinoma (LUAD) (Pan-Cancer Atlas) and TCGA head and neck squamous cell carcinoma (HNSC) (Pan-Cancer Atlas) divided into *KEAP1/NFE2L2* wild type (LUAD: *n* = 417; HNSC: *n* = 449) and *KEAP1/NFE2L2* mutant tumors (LUAD: *n* = 109; HNSC: *n* = 46). Data were downloaded from the GDC portal (https://portal.gdc.cancer.gov (accessed on 8 March 2021)). Statistical analysis was done using the Kruskal–Wallis test (** *p* < 0.01, **** *p* < 0.0001).

NRF2 could regulate these genes in a direct or an indirect manner. To reveal the mode of gene regulation, we revisited NRF2-ChIP sequencing data performed with UACC-62 cells in the presence of the NRF2 inducer sulforaphane [23]. A specific NRF2 binding peak was detected in the promoter region of the *EGF* gene (Figure 2A, red arrow). The center of the binding region contains a canonical antioxidant response element (ARE), which is similar to the AREs of bona fide NRF2 target genes *NQO1*, *HMOX1*, and *SLC7A11* and is located at position -19, in close proximity to the *EGF* transcriptional start site (Figure 2B). Furthermore, *EGF* is induced by oxidative stress generated by H_2_O_2_ treatment (Figure 2C). As these data suggest that *EGF* is a canonical NRF2 target, we analyzed *EGF* gene expression in TCGA databases of lung adenocarcinoma (LUAD) and head and neck squamous cell carcinoma (HNSC), where NRF2 is constitutively activated in approximately 23% and 13% of cases, respectively, due to mutations in *KEAP1* and *NFE2L2* [36,37]. Indeed, *EGF* expression was significantly elevated in *KEAP1/NFE2L2*-mutated tumors of both entities compared to *KEAP1/NFE2L2* wild type tumors (Figure 2D).

In summary, NRF2 enhances EGFR activity by inducing the expression of *EGFR* and its ligands in melanoma, with *EGF* transcription being directly regulated by NRF2.

### 2.2. NRF2-Dependent Repression of MITF Activity Mediates EGFR and TGFA Expression

**Figure 3:**
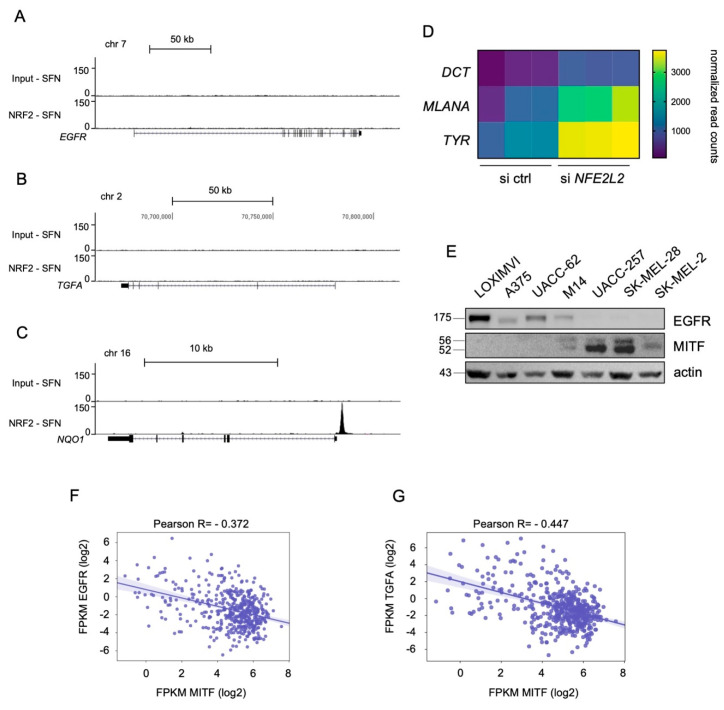
**Negative correlation of MITF and EGFR/TGFA.** (**A**–**C**) Genome browser tracks of the *EGFR* (A), *TGFA* (B), and *NQO1* (C) genomic regions after NRF2 ChIP-Seq analysis [23]. NRF2 was stabilized by treatment with sulforaphane (7.5 µM SFN, 24 h). (**D**) Differential expression of MITF target genes in UACC-62 melanomas cells treated with control or *NFE2L2*-specific siRNA. Data are derived from previously published transcriptome data [23] (Bioproject accession number PRJNA601317) and normalized read counts were analyzed in GraphPadPrism (padj < 0.05). (**E**) Immunoblot of EGFR and MITF protein levels in indicated melanoma cell lines. Actin served as loading control. (**F**,**G**) Linear regression analysis of *MITF* and *EGFR* mRNA (F) or *MITF* and *TGFA* mRNA (G) (*n* = 470). The results shown here are based upon data derived from the TCGA dataset Skin Cutaneous Melanoma, and FPKM values were downloaded from the GDC portal (https://portal.gdc.cancer.gov (accessed on 8 March 2021)).

In contrast to *EGF*, we did not detect an enrichment of NRF2 in the promoter regions of either *EGFR* or *TGFA* (Figure 3A,B). The distinct NRF2 peak in the promoter region of *NQO1* served as positive control (Figure 3C). *EGFR* and *TGFA* are therefore likely regulated by NRF2 in an indirect manner.

We previously reported that NRF2 is a strong suppressor of MITF activity and differentiation in melanoma and represses transcriptional activity of MITF [23]. Consequently, MITF targets and differentiation genes *DCT*, *MLANA*, and *TYR* are upregulated in UACC-62 cells with NRF2 knockdown, as detected by RNA sequencing (Figure 3D). *MITF* and *EGFR* are expressed in melanoma cells in a mutually exclusive manner [12] (Figure 3E, Supplementary Figure S1), and MITF was described previously to suppress *TGFA* expression [19]. In the TCGA SKCM dataset, *EGFR* as well as *TGFA* are negatively correlated to *MITF* (Figure 3F,G). Thus, we hypothesized that NRF2 enhances *EGFR* and *TGFA* expression indirectly via MITF suppression.

**Figure 4:**
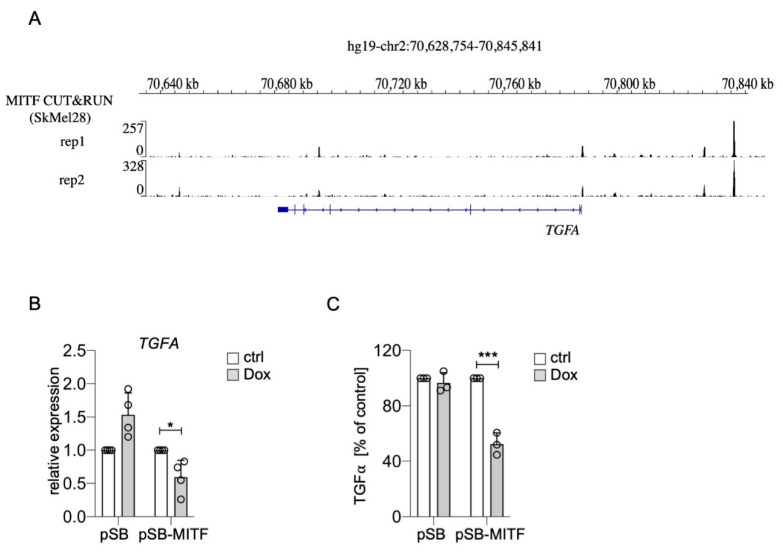
**Suppression of *TGFA* by MITF.** (**A**) Genome browser tracks of the *TGFA* genomic region after MITF ChIP-Seq analysis of SK-MEL-28 cells [40]. (**B**) Real-time PCR of *TGFA* gene expression in UACC-62 cells transgenic for the pSB control vector or the doxycycline-inducible pSB-MITF vector (500 ng/mL Dox, 3 d). (**C**) TGFα secretion, measured by ELISA, from UACC-62-pSB and pSB-MITF cells as described in subfigure (B). For subfigures (B,C), one-way ANOVA with Dunnett’s multiple comparisons test was carried out to calculate significant differences (* *p* < 0.05, *** *p* < 0.001). Error bars represent SD.

**Figure 5:**
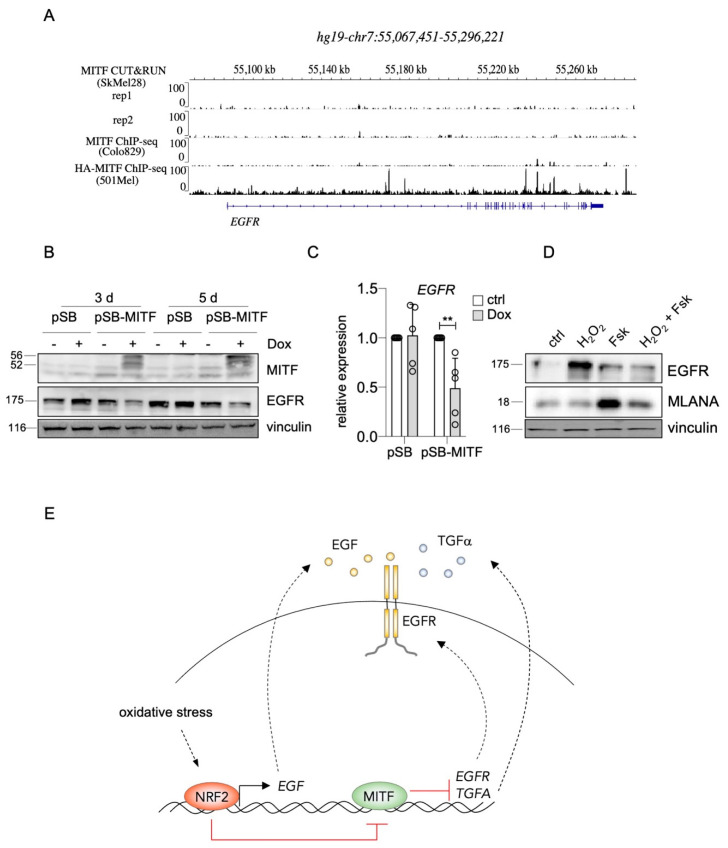
**Suppression of *EGFR* by MITF.** (**A**) Genome browser tracks of the *EGFR* genomic region after MITF ChIP-Seq analysis of indicated melanoma cell lines [40]. (**B**) Immunoblot of MITF and EGFR expression after Dox-inducible expression of MITF for 3 d. Vinculin served as loading control. (**C**) Real-time PCR of *EGFR* gene expression in UACC-62 cells transgenic for the pSB control vector or the doxycycline-inducible pSB-MITF vector (as in subfigure (B)). (**D**) Western blot, demonstrating EGFR and MLANA protein levels in UACC-62 cells treated, where indicated, with forskolin (“Fsk”, 20 µM, 24 h) and with H_2_O_2_ (400 µM, last 5 h before harvesting). MLANA served as indicator of MITF activity. Vinculin was used as loading control. For subfigure (C), one-way ANOVA with Dunnett’s multiple comparisons test was carried out to calculate significant differences (** *p* < 0.01). Error bars represent SD. (**E**) Overview of the proposed mechanism of NRF2-dependent EGFR pathway regulation in melanoma. Stress-induced NRF2 binds to the ARE in the *EGF* promoter and leads to elevation of soluble EGF, while simultaneously blocking MITF activity, resulting in derepression of *EGFR* and *TGFA*. This leads to EGFR activation on multiple levels, supporting the maintenance of EGFR^high^/MITF^low^/melanoma cells.

Interestingly, it was recently shown that *TGFA* belongs to the genes most strongly upregulated in SK-MEL-28 MITF-knockout melanoma cells compared to their controls. Furthermore, the authors performed MITF CUT&RUN ChIP-sequencing analysis, and genome browser tracks of this dataset reveal distinct MITF peaks in the *TGFA* genomic region including the promoter (Figure 4A) [40]. When we increased MITF levels in UACC-62 melanoma cells with a doxycycline-inducible expression vector, *TGFA* was repressed on RNA level, and TGFα secretion, measured by ELISA, was clearly reduced (Figure 4B,C). For *EGFR*, ChIP sequencing analyses revealed multiple MITF binding sites throughout the EGFR genomic region in the melanoma cell line 501Mel and few binding sites in Colo829 and SK-MEL-28 melanoma cells (Figure 5A), implying a possible direct role of MITF in *EGFR* expression regulation. Indeed, MITF overexpression in UACC-62 cells led to the reduction of EGFR on protein as well as RNA level (Figure 5B,C). When we treated melanoma cells with forskolin, a strong stimulator of the cAMP pathway that leads to MITF activation as visible by MLANA expression, the H_2_O_2_-mediated upregulation of EGFR was prevented (Figure 5D). Interestingly, in presence of H_2_O_2_, forskolin-dependent induction of MLANA was reduced—consistent with the suppressive effect of NRF2 on MITF—but was still higher compared to the controls.

In contrast, expression of *EGF* did not correlate with *MITF* in SKCM melanomas (Supplementary Figure S2A), and artificial *MITF* overexpression had no effect on *EGF* expression (Supplementary Figure S2B).

In conclusion, enhanced *EGFR* and *TGFA* expression by NRF2 is at least in part mediated by derepression caused by the inhibitory effect of NRF2 on MITF. The presented data led to the model shown in Figure 5E. Stress-induced NRF2 binds to the ARE in the *EGF* promoter region and causes the secretion of EGF, which can stimulate EGFR-positive melanoma cells in an auto- and paracrine manner. In addition, by inhibiting MITF, NRF2 leads to the derepression of *EGFR* and *TGFA*, triggering EGFR activation even more.

### 2.3. NRF2 Plays a Major Role in EGFR Signaling

**Figure 6:**
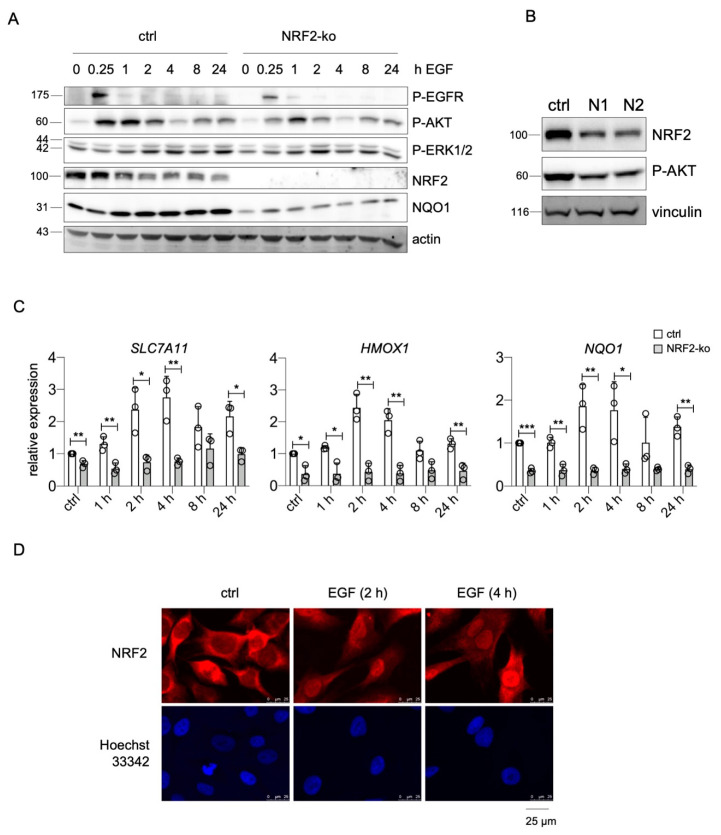
**Cross-activation of NRF2 and EGFR pathways.** (**A**) Protein blot, showing the activation of P-EGFR (Tyr1173), P-AKT (Ser473), and P-ERK1/2 (Thr202/Tyr204) as well as NRF2 and NQO1 expression in UACC-62 controls and UACC-62-NRF2-ko cells after indicated times of EGF stimulation (100 ng/mL). Actin served as loading control. (**B**) Western blot of NRF2 and P-AKT (Ser473) after transfection with control or *NFE2L2*-specific siRNA (3 d). Vinculin served as loading control. (**C**) Real-time PCR of NRF2 pathway target genes *SLC7A11*, *HMOX1*, and *NQO1* in UACC-62 controls and UACC-62-NRF2-ko cells after indicated times of EGF stimulation (100 ng/mL). Two-tailed Student’s *t*-test test was carried out to calculate significant differences (* *p* < 0.05, ** *p* < 0.01, *** *p* < 0.001). Error bars represent SD. (**D**) Immunofluorescence of NRF2 in UACC-62 cells and corresponding nuclear staining (Hoechst 33342) after indicated times of EGF induction (100 ng/mL).

The presented data imply that endogenous NRF2 constantly supports EGFR activity by elevating the levels of both receptor and ligands. To investigate if NRF2 also affects EGFR signaling in response to external growth factors, we used UACC-62 NRF2-ko cells that were treated for different timepoints with EGF and compared their cellular responses to those of UACC-62 cells. We examined the levels of phosphorylated ERK1/2 and AKT, which are activated downstream of EGFR, and monitored phosphorylated EGFR as well as NRF2. Basal ERK1/2 phosphorylation was rather high even before EGF treatment, which is expected due to the presence of the endogenous BRAF^V600E^ mutation in this cell line. Activation of the EGFR pathway led to a slightly enhanced ERK1/2 phosphorylation after 2 h or more. However, the presence of NRF2 had no major effect on P-ERK1/2 levels (Figure 6A). In contrast, P-AKT, indicating PI3K pathway activity, was transiently increased after EGF stimulation. NRF2-ko cells showed a reduced extent of AKT phosphorylation compared to the controls. Notably, a reduced basal AKT phosphorylation is also detected when NRF2 is knocked down by siRNA (Figure 6B). Furthermore, NRF2-ko cells showed reduced EGFR phosphorylation (Figure 6A), indicating that NRF2 is important for full EGFR pathway activity in melanoma. In addition, we noticed that EGFR activation led to an increase in NQO1 protein only in presence of NRF2 (Figure 6A). As *NQO1* belongs to the bona fide target genes of NRF2 and an EGFR-mediated induction of NRF2 was reported previously, the effect of EGF stimulation on the NRF2 target genes *NQO1* as well as *SLC7A11* and *HMOX1* was investigated. Indeed, EGF caused the induction of all three genes in an NRF2-dependent manner (Figure 6C). NRF2-specific immunofluorescence further confirmed the positive effect of EGF on NRF2, as EGF treatment led to a profound nuclear translocation of NRF2 (Figure 6D).

In summary, NRF2 initiates a mechanism that leads to the maintenance of melanoma cells with high EGFR and low MITF levels, where NRF2 and EGFR pathways enhance each other in a positive feedback loop.

## 3. Discussion

The expression of various receptor tyrosine kinases, including AXL, PDGFRB, and EGFR, in cutaneous melanoma occurs in MITF^low^ melanoma cells with a proinvasive potential and has been associated with BRAF/MEK inhibitor resistance [3,12,18,19,41,42,43]. Data from non-small cell lung cancer (NSCLC) also imply a role of EGFR in resistance towards immune checkpoint inhibitor therapy [44]. In contrast to NSCLC, EGFR is not mutated in melanoma, but is rather induced, e.g., as a result from therapy stress. This regulation was described to be dependent on post-translational as well as transcriptional mechanisms. At the post-translational level, tyrosine kinase ACK1, which is downregulated in some BRAF inhibitor-resistant melanomas, induces EGFR turnover [45]. At the transcriptional level, *EGFR* expression has been connected to the activity of the transcription factor sex-determining region Y-box 10 (SOX10) [43]. Here, suppression of SOX10 leads to the elevation of transforming growth factor β (TGFβ), which causes a profound upregulation of EGFR. In contrast to SOX10, RUNX2 can increase the expression of several receptor tyrosine kinases including EGFR in melanoma [46]. These data already point to a strong inverse correlation between differentiation and EGFR in melanoma, as SOX10 is a neural crest transcription factor that is crucial for melanocytic fate and one of the main inducers of MITF [47]. RUNX2, on the other hand, was recently described to be suppressed by MITF [48], while MITF itself efficiently reduces EGFR levels—as shown in the present study—and limits the expression of the EGFR ligands TGFA and HB-EGF [19,40]. Consequently, overexpression of MITF results in decreased EGFR activity, as demonstrated by reduced phosphorylation of the receptor [19].

Here, we demonstrate that NRF2 has a similar effect, as it is an efficient suppressor of MITF activity [23], and thus NRF2 activation results in the derepression of *EGFR* and *TGFA*. MITF ChIP sequencing data from previous studies show that MITF binds to the genomic regions of *TGFA* and *EGFR*, indicating that they might be direct targets of MITF. Due to the specific expression of MITF in the melanocytic lineage, this indirect MITF-dependent effect of NRF2 is typical for melanoma but cannot occur in other cancer or tissue types. In contrast, ChIP sequencing data showed that NRF2 specifically binds to the *EGF* promoter via an antioxidant response element and directly induces *EGF* expression. The *EGF* ARE sequence was recently described as an NRF2 target in luciferase reporter assays [49], demonstrating that the effect of NRF2 on EGF should be independent of the cell lineage. Indeed, *EGF* expression correlated with NRF2 activation in different cancer types, as we showed for *NFE2L2/KEAP1* mutant lung adenocarcinoma as well as head and neck squamous cell carcinoma.

An EGFR-potentiating function of NRF2 should therefore be universal and was indeed described in some studies using either mutant or endogenous NRF2. However, the underlying EGFR activation mechanisms were reported differently. The group of Michael Karin found an increase in several EGFR ligands including *EGF* after mutationally activated NRF2 in a mouse model of hepatomegaly [34] as well as in human pancreatic cancer cells with ATG7 deletion [49]. In a murine model for pancreatic cancer, the authors attributed the observed increased EGF secretion of organoid tumor cultures to ligand shedding, mediated by NRF2-dependent stimulation of the ADAM10 metalloprotease [50]. As EGFR pathway activation is so strongly connected to BRAF/MEK inhibitor resistance in melanoma, it is likely that stress-induced NRF2 activation during therapy significantly contributes to this effect. Future studies will reveal the relative importance of the EGFR ligands EGF and TGFα in this process.

Notably, mutations in *KEAP1/NFE2L2* and those in *EGFR* exclude each other in all cancer types with high somatic mutation rates in these genes, speaking for a high functional overlap of both pathways [51]. In fact, there is significant crossover between the pathways, as NRF2 and EGFR can activate each other [51].

The strong NQO1 induction by EGF as well as the NRF2-dependent increase of PI3K pathway activity observed in the present study are examples of this signaling crossover. Further in-depth analyses are required to reveal the full extent of the functional overlap, as this could open up therapeutic windows for tumors with mutationally or stress-activated NRF2 pathway.

## 4. Materials and Methods

### 4.1. Cell Culture

UACC-62, M14, LOXIMVI, UACC-257, and SK-MEL-2 cells were obtained from NCI/NIH (DCTD Tumor Repository, National Cancer Institute at Frederick, Frederick, MD, USA). A375 and SK-MEL-28 cells were received from ATCC. All cells have been authenticated and are regularly tested for contaminations. UACC-62 NRF2-ko cells as well as UACC-62-pSB and -pSB-MITF cells were previously described [23]. Cultivation was done in DMEM with 10% FCS (PAN biotech, Aidenbach, Germany) and 1% penicillin/streptomycin (Sigma-Aldrich, St. Louis, MO, USA) at 37 °C and 5% CO_2_. Where indicated, doxycycline, H_2_O_2_, forskolin (Sigma-Aldrich, St. Louis, MO, USA), or EGF (PeproTech, Rocky Hill, CT, USA) was added. Before EGF treatment or ELISA assay, cells were washed with PBS and starved for 1 day in DMEM medium containing 1% dialyzed FCS (Gibco/Life Technologies, Carlsbad, CA, USA).

### 4.2. RNA Extraction, cDNA Synthesis, and RT-qPCR

RNA isolation of cell pellets was performed using TRIzol reagent (Invitrogen, Carlsbad, CA, USA) according to the manufacturer’s protocol. cDNA synthesis was done using the RevertAid First Strand cDNA Synthesis Kit (Fermentas, Waltham, MA, USA) with hexamer primers in accordance with the manufacturer’s protocol. RT-qPCRs were performed and analyzed with the CFX Connect^TM^ Real-Time System (Bio-Rad Laboratories, Munich, Germany) using SYBR Green reagent. Gene expression was normalized to *ACTB* as housekeeping gene using the ΔΔct method. The sequences of the oligonucleotides purchased from Sigma-Aldrich (St. Louis, MO, USA) are indicated in Supplementary Table S1.

### 4.3. siRNA Transfection

Non-targeting siRNA (Sigma-Aldrich, St. Louis, MO, USA; SIC001) or siRNA directed against human *NFE2L2* (Ambion, Carlsbad, CA, USA, AM16704; ID3347, ID107967) was delivered to the cells using XtremeGene siRNA transfection reagent (Roche, Basel, Switzerland) in accordance with the manufacturer’s instructions. *NFE2L2* siRNA target sequences are shown in Supplementary Table S2.

### 4.4. Protein Lysis and Western Blot

Cells were lysed in RIPA lysis buffer (50 mM Tris pH 8.0; 50 mM NaCl; 1% Nonidet-P40; 0.5% deoxycholate, 0.1% SDS; 10 μg/mL aprotinin; 10 μg/mL leupeptin; 200 μM Na_3_VO_4_; 1 mM PMSF; and 100 mM NaF). Shortly after, 40 μg of protein was separated by 10–12% polyacrylamide SDS-PAGE. Proteins were transferred onto Amersham^TM^ nitrocellulose membranes (GE Healthcare, Chicago, IL, USA) and were blocked with 5% BSA (Serva, Heidelberg, Germany) in TBS with 0.1% Tween-20. Incubation with the primary antibodies took place at 4 °C. The following antibodies were used in this study.

NRF2 ([EP1808Y], ab62352) and MLANA (ab210546) antibodies were purchased from Abcam, Cambridge, UK. Antibodies directed against P-ERK1/2 (Thr202/Tyr204) (9101), P-AKT (Ser473) (#4060), EGFR (D38B1) (#4267), and P-EGFR (Tyr1173) (#4407) were from Cell Signaling Technology, Danvers, USA. Antibodies against NQO1 (sc-32793) and beta-actin (sc-47778, Santa Cruz Biotechnology, Dallas, TX, USA) were purchased from Santa Cruz. Anti-vinculin (V9131) was purchased from Sigma-Aldrich (St. Louis, MO, USA). The MITF antibody was kindly provided by C. Goding (Ludwig Institute for Cancer Research, University of Oxford, Oxford, UK). For protein detection, membranes were incubated with secondary antibodies: anti-mouse (31444) (Thermo Scientific, Waltham, MA, USA) or anti-rabbit (170-6515, Bio-Rad, CA, USA) coupled to horseradish peroxidase and visualized with the ECL detection kit (Thermo Scientific, Waltham, MA, USA) or with the Alexa fluor 488 anti-mouse antibody (A11017) (Invitrogen, Carlsbad, CA, USA) and a Fusion SL imaging system (Vilber Lourmat, Eberhardzell, Germany). The molecular weight of detected proteins is indicated in kDa. Western blot quantifications, related to the respective loading control, are provided in the supplementary information. Protein amounts were quantified using the gel analyzer tool Fiji (ImageJ).

### 4.5. ELISA Assay

To measure EGF and TGFα, UACC-62 melanoma cells were seeded into 6 cm plates and were treated as indicated. After 3 days, supernatant was analyzed with the human TGFα (DTGA00) or the human EGF (DEG00) Quantikine ELISA Kit (R&D Systems, Minneapolis, MN, USA) in accordance with the manufacturer’s instruction. Absorbance at 450 nm was measured with a microplate reader (FLUOstar^®^ Omega, BMG Labtech, Ortenberg, Germany).

### 4.6. Immunofluorescence

NRF2 immunofluorescence was done as described previously [23]. As primary antibody, anti-NRF2 (ab62352, Abcam, Cambridge, UK) was used. After three PBS washing steps, secondary antibody incubation was carried out in the dark for 1 h at room temperature with Alexa Fluor^®^ 594 goat anti-rabbit IgG (11037) (Life Technologies, Carlsbad, CA, USA). Hoechst 33342 (Invitrogen, Carlsbad, CA, USA) was used for nuclear staining. Samples were analyzed by inverted fluorescent microscopy (Leica, Wetzlar, Germany).

## Data Availability

Not applicable.

## Supplementary Materials

The following material is available online at https://www.mdpi.com/article/10.3390/ijms22083803/s1, Supplementary Figure S1: Negative correlation of EGFR and MITF expression in melanoma, Supplementary Figure S2: No effect of MITF on EGF expression in melanoma; Supplementary Table S1: Human oligonucleotides used for RT-qPCR, Supplementary Table S2: siRNA sequences used in this study, quantified and uncropped western blot images.
