# Supplementary material for: NRF2 Enables EGFR Signaling in Melanoma Cells
Source: Int J Mol Sci. 2021 Apr 7;22(8):3803. doi: 10.3390/ijms22083803 (PMC8067606; doi:10.3390/ijms22083803)

Supplementary information

Supplementary Figures

Supplementary Figure 1

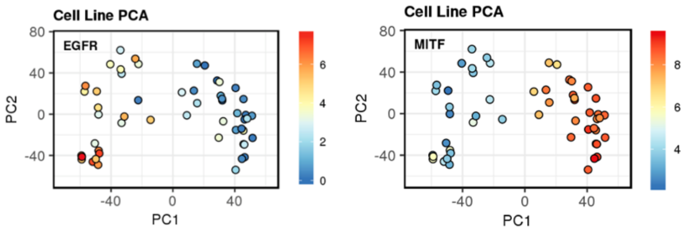

Supplementary Figure 1: Negative correlation of EGFR and MITF expression in melanoma
Melanoma gene expression color maps, showing the expression of EGFR and MITF in 53 melanoma cell lines, visualized in the context of principal component analysis of RNA expression data. Data are derived from https://systems.crump.ucla.edu/dediff/index.php.

Supplementary Figure 2

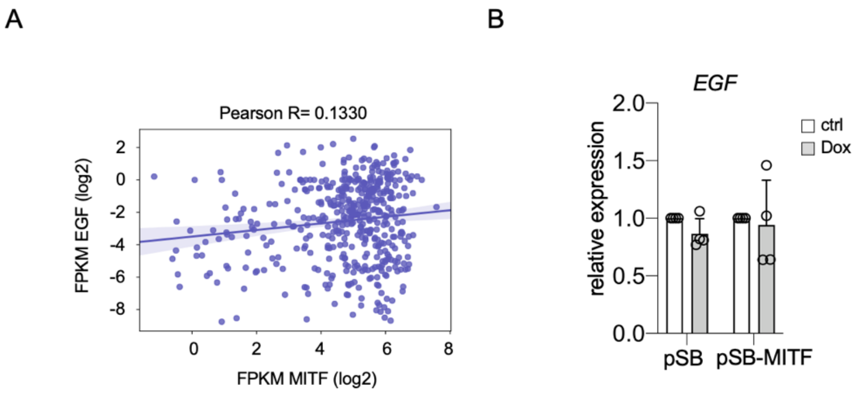

Supplementary Figure 2: No effect of MITF on EGF expression in melanoma
A: Linear regression analysis of *MITF* and *EGF* mRNA (n = 470). The results shown here are based upon data derived from the TCGA dataset Skin Cutaneous Melanoma, and FPKM values were downloaded from the GDC portal (https://portal. gdc.cancer.gov). **B:** Real-time PCR of *EGFR* gene expression in UACC-62 cells transgenic for the pSB control vector or the doxycycline-inducible pSB-MITF vector (3 d).

Supplementary Tables

Supplementary Table 1

Human oligonucleotides used for RT-qPCR

Gene	ENSEMBL ID	Forward Primer	Reverse Primer
ACTB	ENSG00000075624	5´-GGCATCCTGACCCTGAAGTA-3´	5´-GGGGTGTTGAAGGTCTCAAA-3´
EGF	ENSG00000138798	5´-TGACTCTACTCCACCCCCTCACCT-3´	5´-AGGTCTCGGTACTGACATCGCTCC-3´
EGFR	ENSG00000146648	5´-AGTGCTGGATGATAGACGCA-3´	5´-CCTGAATGACAAGGTAGCGC-3´
HMOX1	ENSG00000100292	5´-CTTCTTCACCTTCCCCAACA-3´	5´-AGCTCCTGCAACTCCTCAAA-3´
NQO1	ENSG00000181019	5´-AGCCCAGATATTGTGGCTGA-3´	5´-CGGAAGGGTCCTTTGTCATA-3´
SLC7A11	ENSG00000151012	5´-TTTGCACCCTTTGACAATGA-3´	5´-GGAAAACAAAGCTGGGATGA-3´
TGFA	ENSG00000163235	5´-TCGCTCTGGGTATTGTGTTG-3´	5´-GGGAATCTGGGCAGTCATTA-3´

Supplementary Table 2

siRNA sequences used in this study

siRNA	Target sequence
si*NFE2L2*_1 (N1)	5'-GGAGAAAAUGACAAAAGCtt-3'
si*NFE2L2*_2 (N2)	5'-GGAGCUAUUAUCCAUUCCUtt-3'

Quantified western blots

Figure 1

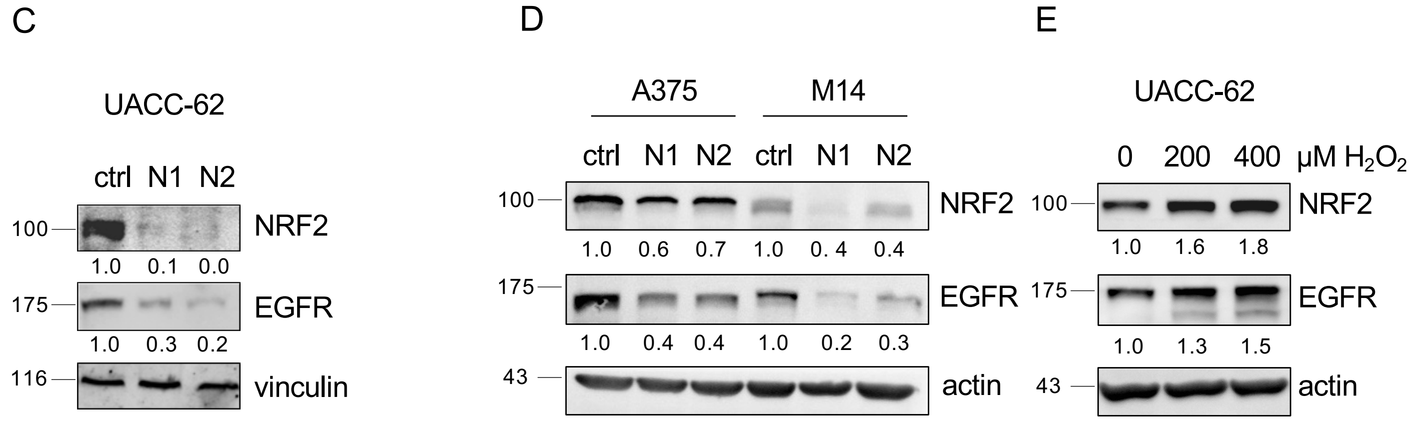

Figure 3

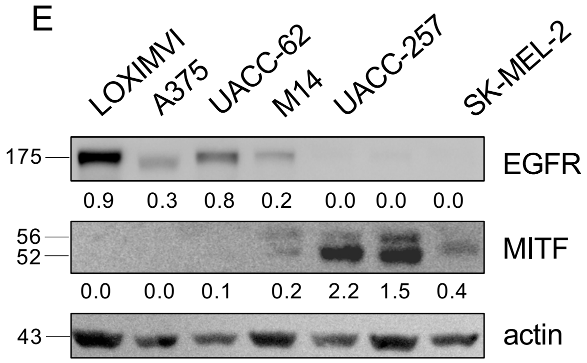
E
LOXIMVI
A375
UACC-62
M14
UACC-257
SK-MEL-2
175
EGFR
0.9 0.3 0.8 0.2 0.0 0.0 0.0
56
52
MITF
0.0 0.0 0.1 0.2 2.2 1.5 0.4
43
actin

Figure 5

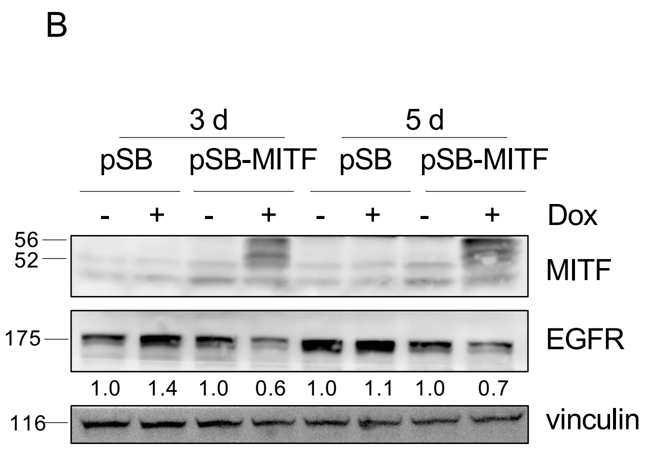
B
3 d
5 d
pSB
pSB-MITF
pSB
pSB-MITF
- + - + - + - +
Dox
56
52
MITF
175
EGFR
1.0 1.4 1.0 0.6 1.0 1.1 1.0 0.7
116
vinculin

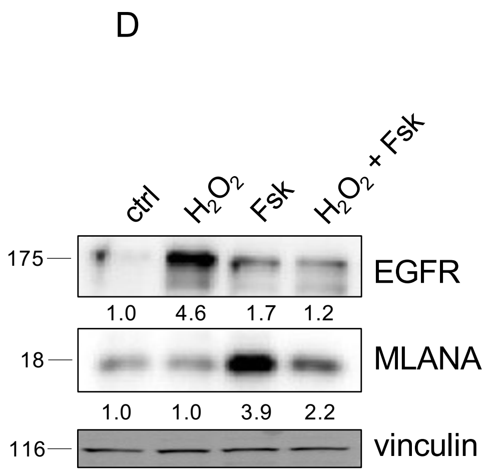
D
ctrl
H2O2
Fsk
H2O2 + Fsk
175
EGFR
1.0 4.6 1.7 1.2
18
MLANA
1.0 1.0 3.9 2.2
116
vinculin

Figure 6

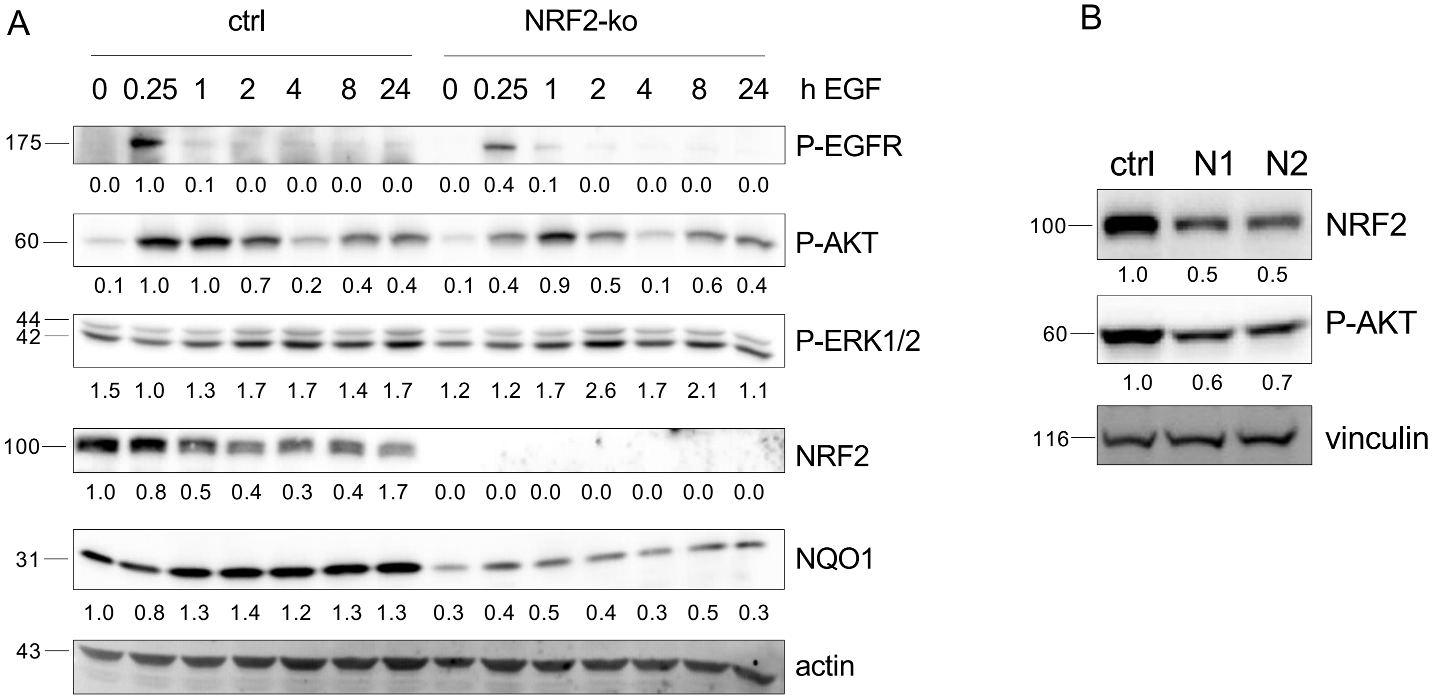
A
ctrl
NRF2-ko
0 0.25 1 2 4 8 24 0 0.25 1 2 4 8 24 h EGF
175
P-EGFR
0.0 1.0 0.1 0.0 0.0 0.0 0.0 0.0 0.4 0.1 0.0 0.0 0.0 0.0
60
P-AKT
0.1 1.0 1.0 0.7 0.2 0.4 0.4 0.1 0.4 0.9 0.5 0.1 0.6 0.4
44
42
P-ERK1/2
1.5 1.0 1.3 1.7 1.7 1.4 1.7 1.2 1.2 1.7 2.6 1.7 2.1 1.1
100
NRF2
1.0 0.8 0.5 0.4 0.3 0.4 1.7 0.0 0.0 0.0 0.0 0.0 0.0 0.0
31
NQO1
1.0 0.8 1.3 1.4 1.2 1.3 1.3 0.3 0.4 0.5 0.4 0.3 0.5 0.3
43
actin
B
ctrl N1 N2
100
NRF2
1.0 0.5 0.5
60
P-AKT
1.0 0.6 0.7
116
vinculin

Uncropped Western blot images

Figure 1C

EGFR

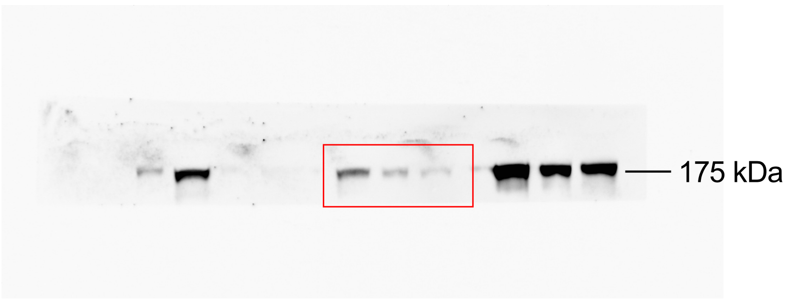

175 kDa

NRF2

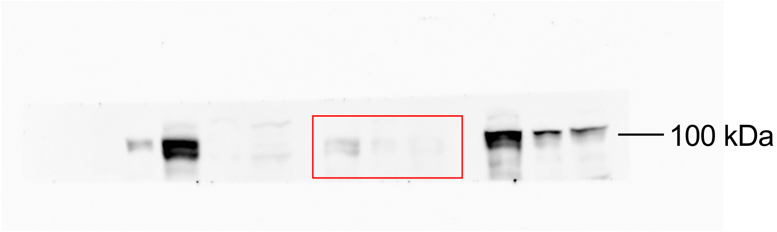

100 kDa

NRF2,
longer exposure

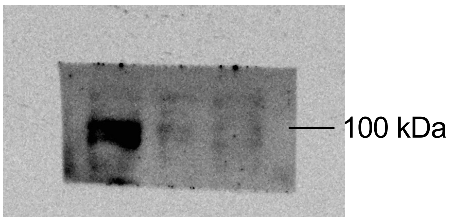

100 kDa

vinculin

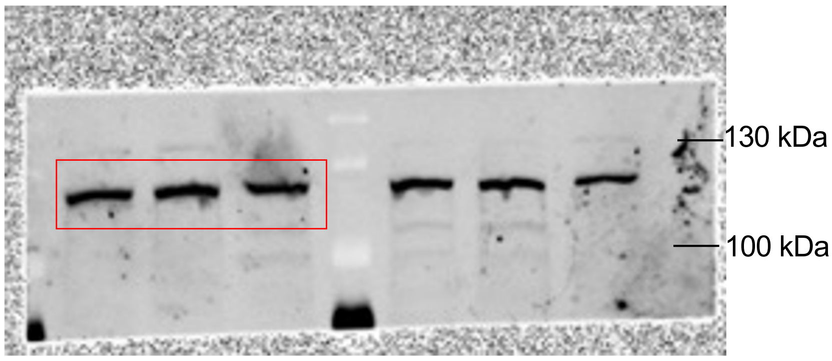

130 kDa

100 kDa

Figure 1D

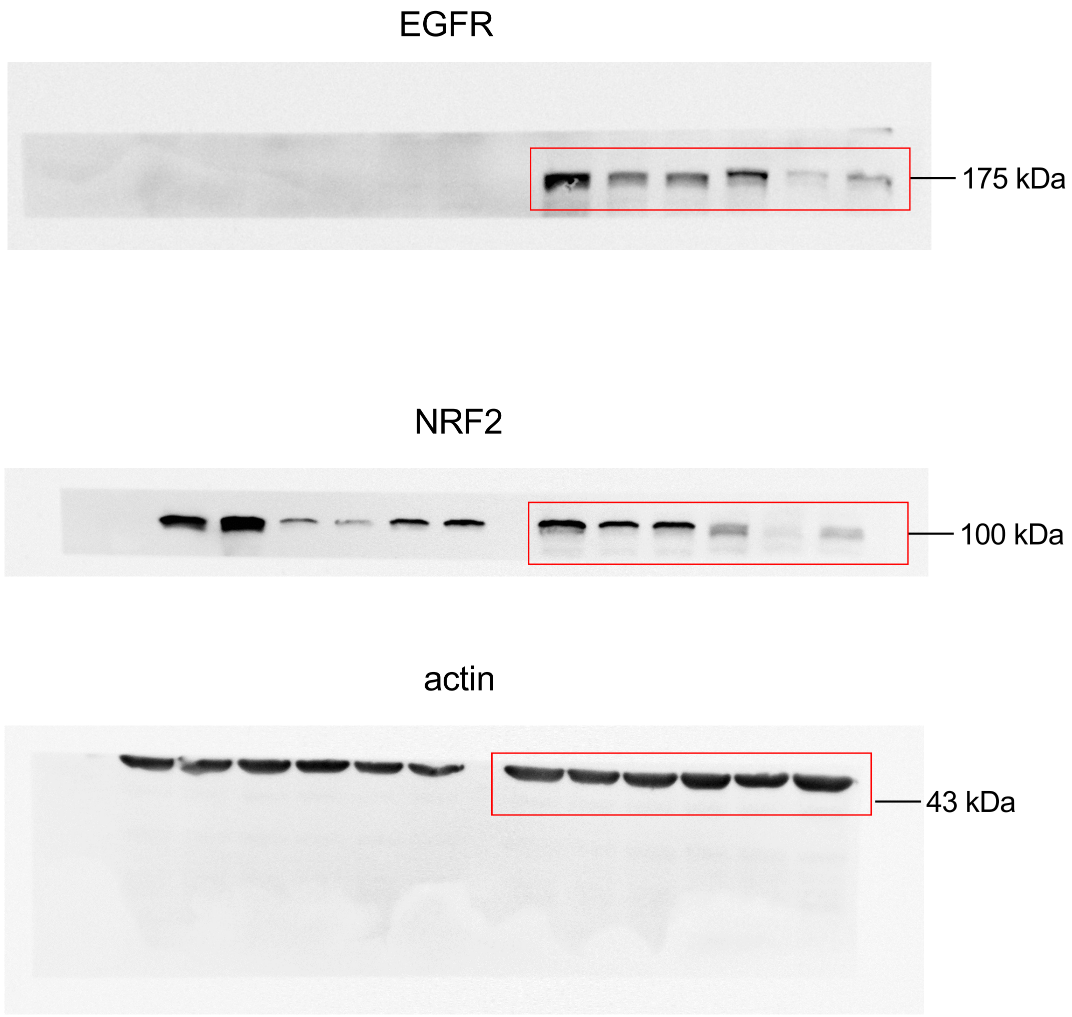

Figure 1E

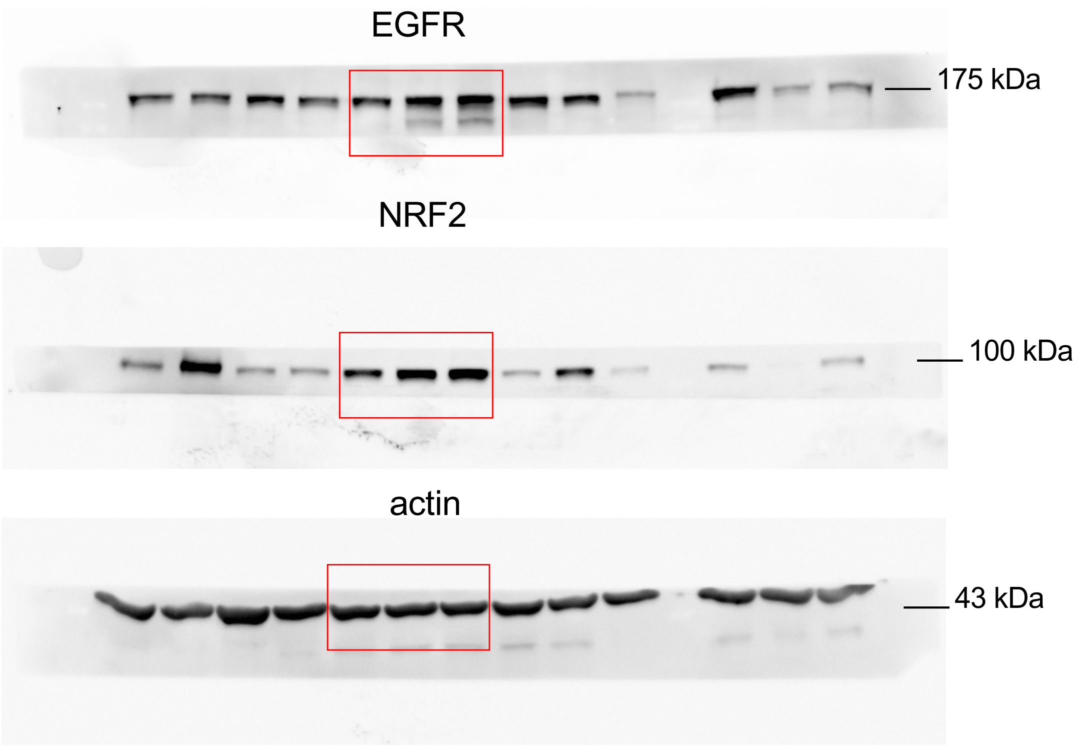

Figure 3E

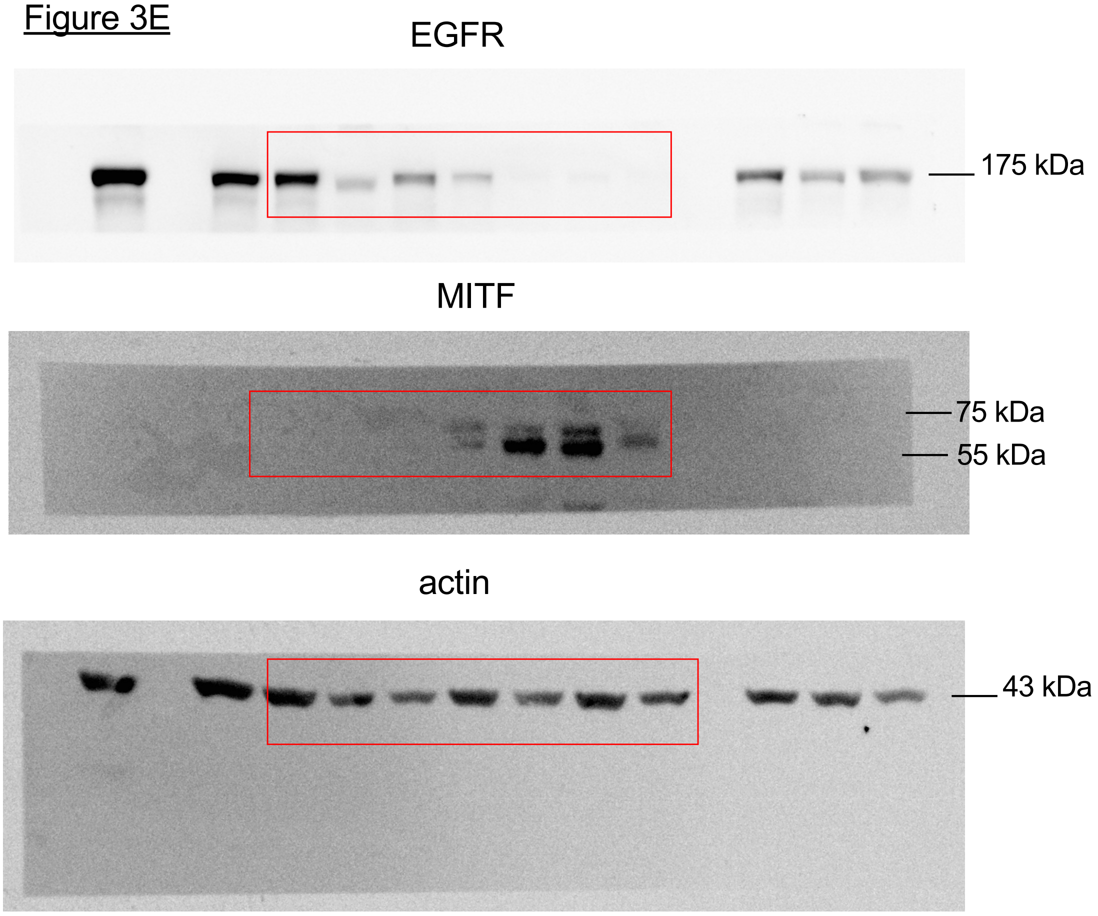

Figure 5B

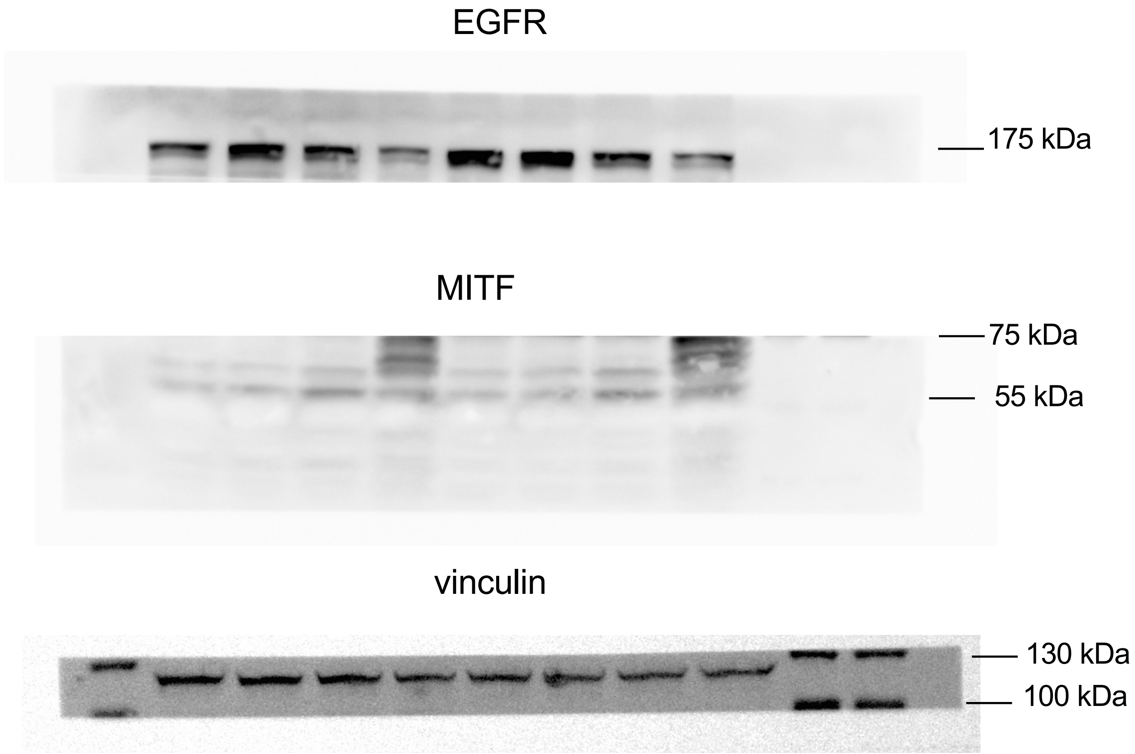

Figure 5D

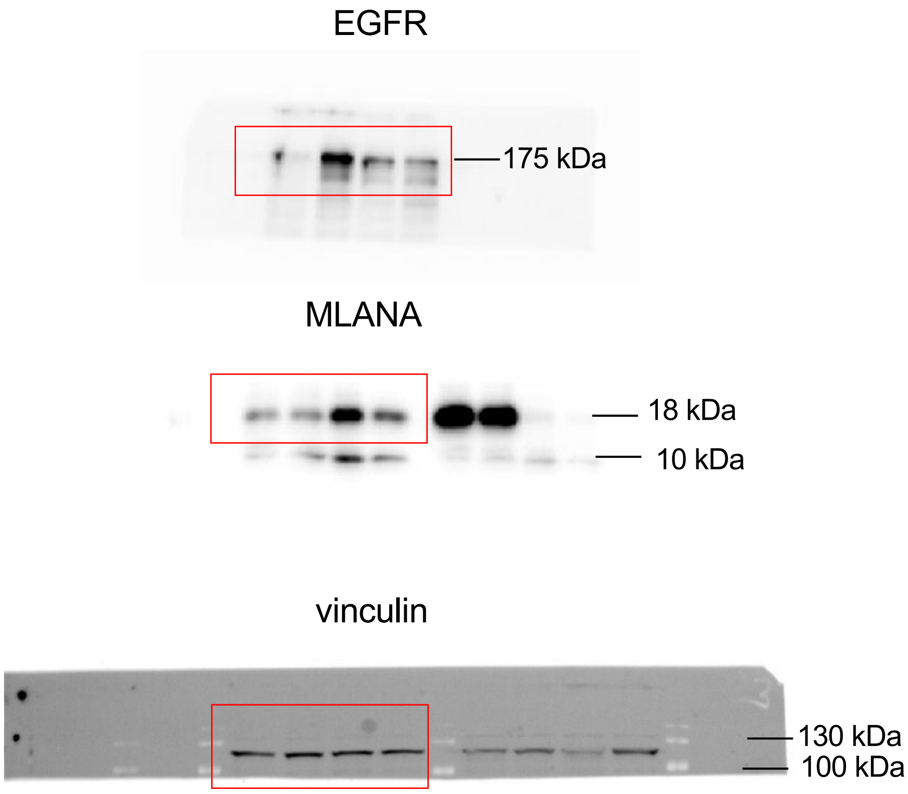

Figure 6A

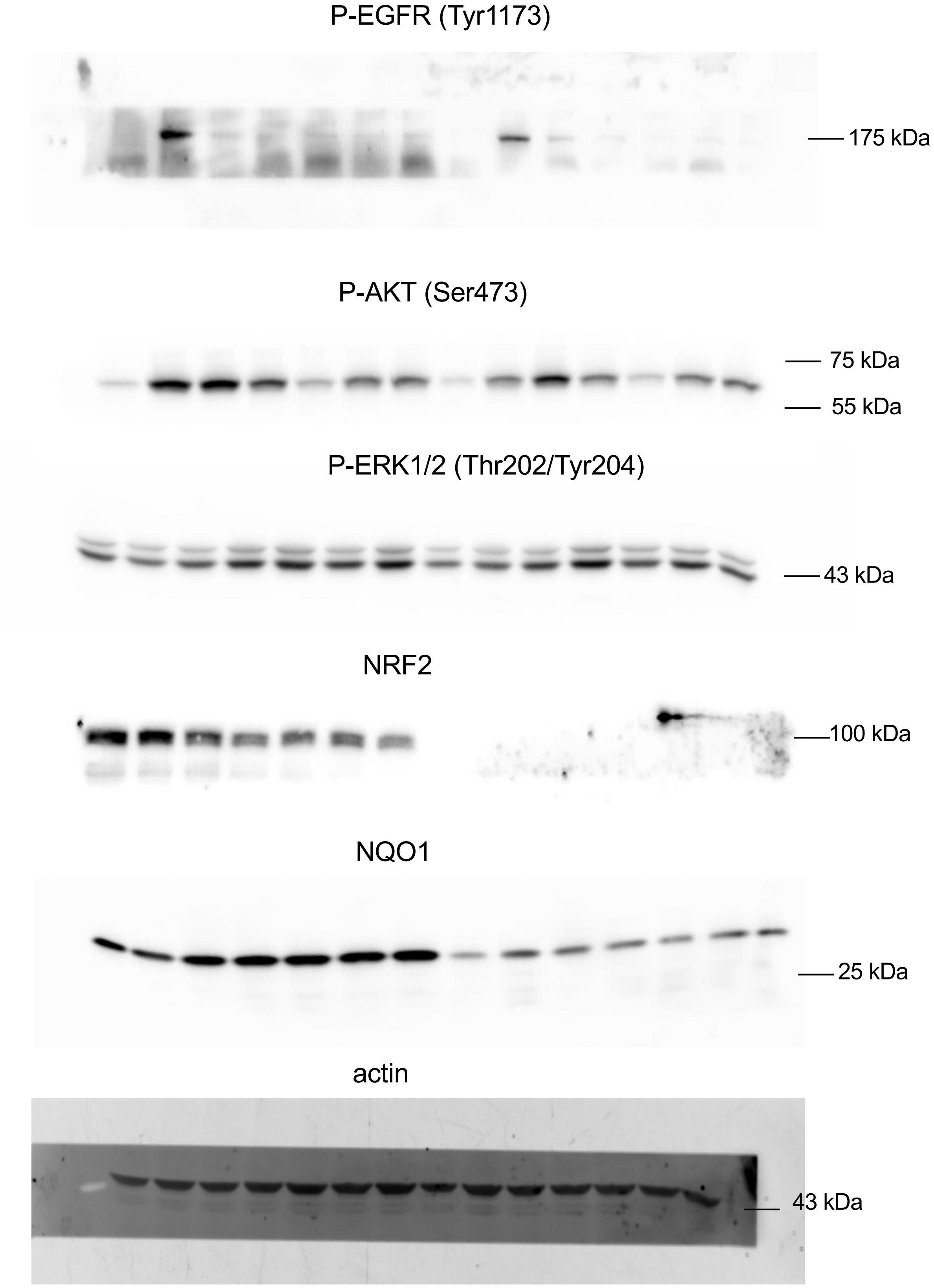

P-EGFR (Tyr1173)
175 kDa
P-AKT (Ser473)
75 kDa
55 kDa
P-ERK1/2 (Thr202/Tyr204)
43 kDa
NRF2
100 kDa
NQO1
25 kDa
actin
43 kDa

Figure 6B

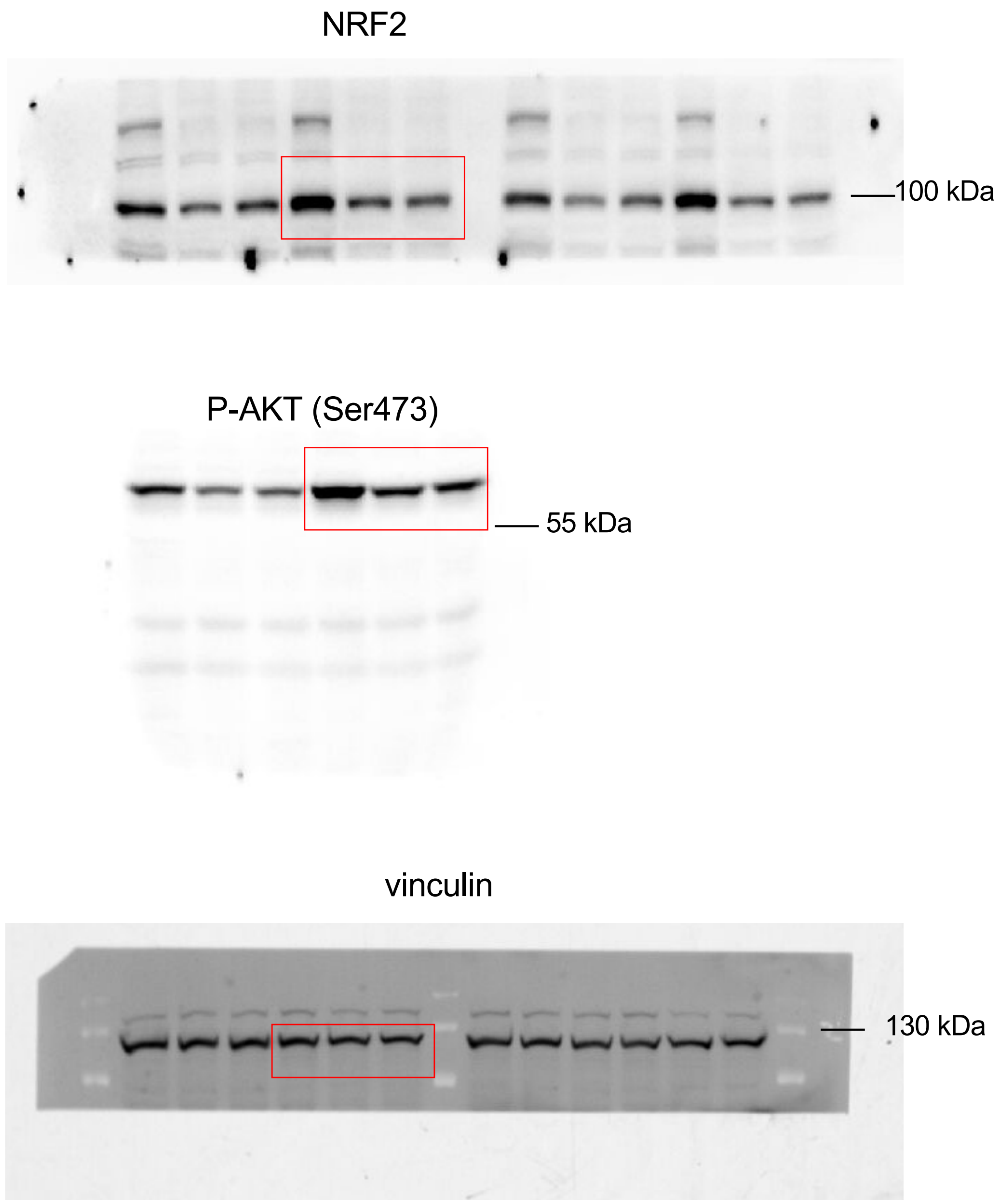

Supplement: Supplementary file 1 [file ijms-22-03803-s001.pdf]
